# Differential Nap‐To‐Nap Stability of Sleep Spindles, Slow Waves, and their Temporal Coupling: An Exploratory Study

**DOI:** 10.1111/jsr.70253

**Published:** 2025-11-25

**Authors:** Damiana Bergamo, Antonino Visalli, Angie Baldassarri, Nicola Cellini

**Affiliations:** ^1^ Department of General Psychology University of Padova Padova Italy; ^2^ Department of Biomedical, Metabolic and Neural Sciences University of Modena Modena Italy

**Keywords:** nap, NREM, NREM fingerprints, sleep spindles, slow waves, spindle‐slow wave coupling

## Abstract

Slow waves and sleep spindles characterise non‐rapid eye movement (NREM) sleep and support cognitive and plasticity‐related functions. While their stability across nights is well established, less is known about their consistency across daytime naps. Nineteen healthy young adults (20–27 years) underwent two 90‐min afternoon naps, interleaved by 1 week, under polysomnographic recording. Slow waves and sleep spindles were detected using standardised algorithms, and their key features, as well as NREM spectral power, were extracted from N2 sleep and assessed for nap‐to‐nap reliability using intraclass correlation coefficients. Temporal coupling between spindles and slow wave events was also evaluated. Spectral power showed good consistency in the sigma band and moderate consistency in the delta band. Spindle frequency, density, and duration were highly reliable, particularly for fast spindles. Slow wave density and slope showed moderate stability, while amplitude and duration were less stable and not consistent, respectively. The phase of slow wave–spindle coupling did not show consistency between naps. Overall, some specific features of NREM sleep, particularly those related to spindles, appear relatively stable across naps and may reflect trait‐like aspects of individual sleep physiology. In contrast, coupling dynamics appear more variable and influenced by state‐related factors.

## 1 Introduction

Non‐rapid eye movement (NREM) sleep is characterised by two key oscillatory phenomena—slow waves and sleep spindles—which represent main markers of sleep‐dependent plasticity (Diekelmann and Born 2010; Tononi and Cirelli 2003). Slow waves reflect alternating patterns of cortical activity, shifting between neuronal silence (down states) and bursts of synchronised firing (up states; Steriade et al. 1993). The occurrence of slow wave events is thought to support memory traces reactivation and redistribution, synaptic homeostasis, and contribute to cellular recovery processes that sustain brain function (Rasch and Born 2013; Riedner et al. 2007; Vyazovskiy and Harris 2013). Sleep spindles, generated by thalamocortical circuits, appear as transient bursts of sigma‐frequency activity and are implicated in synaptic plasticity, learning, and cognitive performance (Fernandez and Lüthi 2020). The mutual coupling of these two graphoelements—specifically, the nesting of sleep spindles within the temporal window of slow‐wave events—is considered essential for the consolidation of newly acquired information (Klinzing et al. 2019; Staresina et al. 2015, 2023).

Evidence from overnight sleep studies demonstrates that sleep spindle characteristics, such as individual peak frequency, scalp topography, and measures of amplitude and density, exhibit high intra‐individual stability across nights, supporting their classification as stable, trait‐like neural markers (Cox et al. 2017; Cross et al. 2025; De Gennaro et al. 2005, 2008; Eggert et al. 2015; Mullins et al. 2025; Purcell et al. 2017; Reynolds et al. 2019). Although less extensively investigated, night‐to‐night stability has also been shown for slow‐wave activity and its topographical distribution (Cox et al. 2018; Israel et al. 2012; Mullins et al. 2025). Such findings suggest that the spatiotemporal characteristics of NREM oscillations may reflect trait‐like features of an individual's sleep architecture (Buckelmüller et al. 2006). At the same time, there is also evidence of day‐to‐day variability influenced by prior wakefulness, circadian phase, neuromodulatory activity, and even individual differences in homeostatic responses, particularly in slow‐wave expression (Achermann and Borbély 2017). In line with this, Gorgoni et al. (2019) in a twin study found that although characteristics of K‐complex‐like slow waves are strongly genetically influenced, their within‐pair similarity is reduced in frontal regions, where their expression is most pronounced, likely due to greater sensitivity to environmental and homeostatic influences.

Overnight recordings have provided important evidence, but they involve long monitoring periods, are more susceptible to artefacts and logistical constraints, and are affected by circadian and homeostatic factors that can complicate the interpretation of the results. Notably, both slow‐wave and spindle activity vary substantially across the night, reducing the reliability of analyses that concatenate whole‐night NREM epochs (Andrillon et al. 2011; Riedner et al. 2007). Daytime naps, by contrast, present an opportunity to investigate NREM sleep, particularly stage N2, which is rich in both slow waves and spindles, under more controlled and less homeostatic‐influenced conditions (Mylonas et al. 2020). Despite these advantages, nap studies remain underrepresented in basic research, though not in clinical practice, likely due to the limited and highly variable sleep duration across individuals, which implies difficulties in achieving adequate sample sizes compared to overnight studies (Németh et al. 2023).

An emerging question, then, is whether the overnight stability observed for spindles and slow waves translates to nap‐to‐nap consistency. Identifying electrophysiological ‘fingerprints’ in short daytime sleep may have practical implications for personalised sleep interventions, as stable nap‐related markers may serve as targets to optimise clinical outcomes (Leong et al. 2022), or improve memory and cognitive performance (Cousins et al. 2019; Mednick et al. 2002). Yet despite its potential, an investigation of sleep spindle and slow wave characteristics across repeated nap sessions remains absent in the literature.

In this context, the present exploratory study focuses on the nap‐to‐nap reliability of N2 spectral power and characteristics of slow waves and spindles, including density, amplitude, and duration, by comparing two daytime naps separated by 1 week in a sample of healthy young adults. Additionally, we explored the consistency of slow wave–spindle interactions, as well as coupling dynamics between delta and sigma frequency bands in naps.

## 2 Methods

### 2.1 Participants

Thirty‐four participants (18 females; age range: 20–28 years) were recruited through university bulletin board announcements and word of mouth. All participants completed a screening session and provided written informed consent to the experiment. No exclusion criteria were applied, except for requiring native proficiency in Italian for task‐related purposes, not relevant to the current investigation. Fourteen participants were excluded due to an inability to sleep during one or both experimental sessions, and one additional participant was excluded due to poor signal quality. The final sample consisted of 19 participants (10 males and 9 females), aged between 20 and 27 years (mean age = 24.26 years, SD = 1.97 years), who were enrolled in a two‐nap protocol study. The study received approval from the Ethics Committee of the Department of Psychology at the University of Padua. The experimental sessions took place in the Sleep Psychophysiology Laboratory of the Department of General Psychology at the University of Padua, where participants took two naps in a sound‐isolated room between 2:00 P.M. and 3:30 P.M. Participants received monetary compensation for their participation.

### 2.2 Experimental Protocol

During the initial screening session, participants were assessed for coffee, cigarette, and alcohol consumption. They also completed the Pittsburgh Sleep Quality Index (PSQI; Curcio et al. 2013), the Beck Depression Inventory‐II (BDI‐II; Lange 2007), the State–Trait Anxiety Inventory Y2 (STAI‐Y2; Spielberger and Gorsuch 1983), and the Reduced Morningness‐Eveningness Questionnaire (MEQ‐r; Natale et al. 2006). Questionnaire results can be found in Table S1a. On the day of the experimental session, participants arrived at the Psychophysiology Laboratory, where they were seated in a room equipped with a computer for filling out questionnaires and completing a memory task, the results of which are outside the scope of the present study. The questionnaires included: the STAI, version Y1 (STAI‐Y1; Spielberger and Gorsuch 1983) to assess state anxiety; the PSQI to evaluate sleep quality; the Samn‐Perelli Fatigue Scale (SPF; Samn and Perelli 1982) to measure perceived fatigue levels, and the Stanford Sleepiness Scale (SSS; Hoddes et al. 1973) to assess momentary alertness at the time of testing. The declarative memory task administered in the two nap sessions was the Fact Learning Task (FLT; Cellini et al. 2019). Participants studied 20 scenarios per session—different across sessions but matched for difficulty and counterbalanced in order across the first and second nap sessions—each presenting three facts about distinct real‐world locations through narrated PowerPoint slides. Memory was assessed with open‐ended questions at three time points: immediately after encoding, after the nap, and 48 h later. Participants had unlimited response time and were instructed to answer all questions. After the task, participants underwent a polysomnography (PSG) setup. Once the setup was complete, participants were asked to lie on the bed. The lights were then turned off, and participants were instructed to sleep. Participants were given a fixed 90‐min time‐in‐bed opportunity; afterward participants were awakened. The same experimental procedure was repeated 1 week later.

### 2.3 EEG Recording and Preprocessing

The polysomnography was conducted according to AASM guidelines (Iber et al. 2007). Nine channels (F3, Fz, F4, C3, C4, P3, P4, O1, and O2) were used to record EEG activity and placed according to conventional 10–20 locations. Additionally, two electrodes were placed over the mastoids and used as the reference for subsequent analysis.

The recording reference electrode was positioned at FCz, and the ground electrode at FPz. An additional seven channels were used to monitor peripheral indices. Specifically, three electrodes were used for ECG recording, two were placed under the chin for EMG activity, and two electrodes were used for the EOG. Signal amplification was achieved using the V‐AMP system (Brain Products, GmbH, Munich, Germany), and no filters were applied during electrophysiological recording. Data acquisition was carried out with the open‐source platform OpenVibe (http://openvibe.inria.fr/) at a sampling rate of 256 Hz. For sleep staging, offline band‐pass filters were applied to the EEG, EOG, and EMG signals. Specifically, a 0.3–35 Hz band‐pass filter was used for the EEG and EOG signals, while a 10–100 Hz band‐pass filter was applied to the EMG signal. EEG recordings from the two daytime naps were visually scored by a trained experimenter (D.B.) according to standard criteria (Iber et al. 2007).

The raw EEG signal was then offline preprocessed using custom MATLAB (v. R2021b; The MathWorks Inc., Natick, MA) scripts based on functions from EEGLAB (v. 2022, Delorme and Makeig 2004). First, the continuous EEG signal was band‐pass filtered between 0.1 Hz and 45 Hz. Secondly, an automatic identification of noisy EEG channels was performed. Rejection thresholds were set at > 3 SD for kurtosis, > 2 SD for the spectral test (0.1–40 Hz), and > 3 SD for the probability test. Identified noisy channels were then visually inspected and removed in both nap sessions. The EEG signals from the remaining electrodes were then re‐referenced to the average of the two mastoids and segmented into 5‐s epochs. An automatic detection of bad epochs within N2 and N3 sleep was performed, excluding segments of the EEG signal that exhibited spectral power outside the predefined thresholds of −40 to 40 dB within the frequency range of 4–40 Hz. The amount of time spent in N2 sleep, retained and analyzed for each participant, is reported in Table 1. We focused exclusively on N2 sleep, as this stage is marked by a high density of slow waves and sleep spindles, and was consistently reached by all participants. Indeed, only 12 out of 19 participants reached N3 sleep in both sessions, and among these, time spent in N3 was limited, preventing analysis of this stage.

**TABLE 1:** Sleep architecture and questionnaires.

	Participants	
Group variables	*N*/mean	STD
*N*	19	X
M/F	10/9	X
Age	24.26	± 0.28
Other demographic variables	Mean unit/week	STD unit/week
Coffee	1.45	± 1.05
Cigarettes	0.45	± 0.52
Alcohol	0.69	± 0.47
Screening Questionnaires	Score mean	Score STD
MEQ‐r	11.78	± 3.53
PSQI	5.89	± 2.53
STAI‐Y2	44.36	± 8.51
BDI‐II	10.52	± 5.34

	Nap 1		Nap 2		Stat
	Min (%)/*N*	STD	Min (%)/*N*	STD	
Sleep variables					
SE (%)	60.49%	± 21.47%	76.34%	± 20.29%	*W* = 32 *p* = 0.026*; *r* = 0.52
Wake	36.02 (39.32%)	± 19.23	20.55 (23.06%)	± 17.63	*W* = 155 *p* = 0.325; *r* = 0.55
NREM	53 (57.85%)	± 14.37	62.92 (70.6%)	± 11.55	*W* = 58 *p* = 0.136; *r* = 0.34
SOL	11.61	± 11.95	6.26	± 7.80	*W* = 131 *p* = 0.047*; *r* = 0.48
N2 sleep latency	17.42	± 13.27	9.31	± 8.28	*W* = 139 *p* = 0.019*; *r* = 0.55
N1 (%)	12.81 (13.99%)	± 8.76	8.61 (9.65%)	± 4.48	*t* = 2.04 *p* = 0.055; *d* = 0.47
N2 (%)	28.23 (30.82%)	± 11.47	36.42 (40.87%)	± 16.14	*t* = −1.92 *p* = 0.078; *d* = 0.44
N3 (%)	11.94 (13.04%)	± 12.87	17.89 (20.08%)	± 15.93	W = 46 *p* = 0.148; *r* = 0.35
N2 Slow waves (N per participant)‐Fz	200.76 (7.29 SW/min)	± 129.94	268.05 (7.58 SW/min)	± 154.48	*W* = 49 *p* = 0.325; *r* = 0.42
N2 Spindles (N per participant)‐Fz	137.94 (5.01 spd/min)	± 84.68	203.29 (5.72 spd/min)	± 123.72	*W* = 36 *p* = 0.097; *r* = 0.54

Questionnaires	Score mean	Score STD	Score mean	Score STD	
PSQI	3	± 1.79	3.68	± 2.16	*W* = 38 *p* = 0.215; *r* = 0.32
STAI‐Y1	49	± 1.88	50.15	± 2.06	*W* = 26 *p* = 0.053; *r* = 0.49
SSS	2.73	± 1.04	2	± 0.81	*W* = 92 *p* = 0.009**; *r* = 0.66
Samn–Perelli	3.31	± 1	2.47	± 1.21	*t* = 2 *p* = 0.061; *d* = 0.45

*Note:* Results are presented as mean ± standard deviation (SD) or as density (number of events per minute of N2 sleep). For the number and density of slow waves and sleep spindles, a representative EEG channel was selected (*Fz*). Effect sizes are reported as r‐equivalent (r) for the Wilcoxon signed‐rank tests, and Cohen's d (d) for the *t*‐tests. *p*‐values in bold indicate statistically significant effects prior to multiple‐comparison correction (*p < 0.05; **p < 0.01). Results that remained significant after FDR correction are highlighted in grey.

Abbreviations: BDI‐II: Beck Depression Inventory‐II; MEQ‐R: Morningness–Eveningness Questionnaire (reduced version); PSQI: Pittsburgh Sleep Quality Index; SE: Sleep Efficiency; SOL: Sleep Onset Latency; SSS: Stanford Sleepiness Scale; STAI: State–Trait Anxiety Inventory.

### 2.4 Sleep Architecture and Questionnaire Measures

To assess nap‐to‐nap differences in sleep architecture and questionnaire scores, we compared Nap 1 and Nap 2 using paired‐sample *t*‐tests for normally distributed variables and Wilcoxon signed‐rank tests otherwise. For each comparison, we report *p* values together with effect sizes (Cohen's d for *t*‐tests; r‐equivalent for Wilcoxon tests). Multiple comparisons were adjusted using the False Discovery Rate (FDR, Benjamini and Hochberg, 1995) correction applied separately to sleep variables and questionnaire measures.

### 2.5 N2 Sleep Power Spectrum

To characterise the spectral power distribution during stage N2 sleep, we computed the EEG power spectrum across frontal, central, and parietal electrodes. Occipital electrodes were excluded from this and subsequent analyses, given the low likelihood of this region showing the activity and events of interest. For each participant, EEG data were bandpass filtered between 0.1 and 40 Hz and downsampled to 100 Hz. The continuous recordings were segmented into 30‐s epochs, from which N2 sleep segments were extracted using previously scored hypnograms. After selecting and concatenating N2 epochs, the data were further divided into 7.5‐s non‐overlapping segments. Power spectral density (PSD) estimates were computed using Welch's method, employing a Hamming window equal to the epoch length, no overlap, and a frequency resolution of 0.25 Hz (frequency factor = 4). Mean PSD values across epochs were calculated for all the scalp electrodes. The resulting spectra were integrated using the trapezoidal method over five frequency bands of interest (delta: 0.1–4 Hz, theta: 4–8 Hz, sigma low: 8–13 Hz, sigma high: 13–18 Hz, and broadband: 0.1–40 Hz) to derive absolute band power values. Intraclass correlation coefficients (ICCs) were calculated across nap sessions for each channel and frequency band using a two‐way random‐effects model as implemented in the *ICC* function (Intraclass Correlation Coefficient (ICC),https://www.mathworks.com/matlabcentral/fileexchange/22099‐intraclass‐correlation‐coefficient‐icc, McGraw and Wong 1996). To account for multiple comparisons, the *p* values have been adjusted using the FDR method, and results were considered significant at *q* < 0.05.

### 2.6 Slow Wave Detection

Detection of slow waves in NREM sleep was performed, separately for each channel, using the algorithm described in Riedner et al. (2007). EEG signals from each electrode were band‐pass filtered (0.5–4.0 Hz) using a Chebyshev Type II filter. Only slow waves with a duration of 0.25–1.0 s between consecutive zero crossings and a negative peak amplitude greater than 20 μV were considered. For all the detected slow waves, the following parameters were determined: density (number/min), duration, negative peak amplitude, and negative slope (between the first zero crossing and the negative peak, μV/s). Only slow waves detected during N2 sleep were retained for further analysis. The number of slow waves for each combination of channel, participant, and nap session is reported in Table S1.

### 2.7 Sleep Spindle Detection

Detection of spindles was performed, separately for each channel, using an adaptation of the algorithm described in Mensen et al. (2018). Specifically, a wavelet‐based filter (8–18 Hz) was applied to the EEG time series using a b‐spline wavelet, and the time course of power of the resulting signal was measured by squaring the values and smoothing the time series using a sliding window of 100 ms. Then, potential spindles were defined as points at which power values passed a high threshold corresponding to the median plus 4 times the median absolute deviation (MAD) of signal power. The actual start and end points of identified events were then measured using the crossing times at a second, low threshold corresponding to the median power plus 2 MADs. The thresholds were recomputed for each 30‐s epoch. Only events with a duration between 0.3 and 3 s were retained for further analyses. Finally, a power–ratio threshold was applied to ensure some specificity of the transient power increases within the spindle range. Specifically, for each potential spindle, we computed the ratio of the mean power in the spindle range (8–18 Hz) over the mean power in the neighbouring ranges (6–8 Hz and 18–20 Hz), and we eventually retained the detected event if the obtained value was greater than 1.5.

We employed a broad frequency window of 8–18 Hz for spindle detection to capture both slow and fast sleep spindles and to account for inter‐individual variability. This range partially overlaps with alpha (∼8–12 Hz) and low beta (∼13–18 Hz) activity, but ensures a more comprehensive classification by including slow spindles (8–13 Hz) and fast spindles (13–18 Hz). Our choice is consistent with large‐scale, multicenter studies demonstrating considerable variability in spindle frequency across individuals (Cox et al. 2017; Purcell et al. 2017; Warby et al. 2014). Only N2 sleep spindles were retained for further analyses. The number of spindles for each combination of channel, participant, and nap session is reported in Table S2.

### 2.8 Nap‐To‐Nap Stability of Spindle and Slow Wave Properties

Concerning nap‐to‐nap stability of sleep spindles, we targeted spindle peak frequency (Hz), spindle duration (s), and spindle density (n/min). For all these spindle properties, we tested both fast (≥ 13 Hz) and slow (< 13 Hz) spindles. Concerning nap‐to‐nap stability of slow waves, we examined slow wave negative peak amplitude (μV), duration (s), density (n/min), and negative slope (μV/s).

Nap‐to‐nap stability of spindle and slow wave properties was quantified using intraclass correlation (ICC; two‐way mixed‐effects model, single rating, absolute agreement), computed only for channels with valid data in both nap sessions and with a minimum of 10 detected events per nap. The *p* values have been corrected using the FDR method (*q* < 0.05).

### 2.9 Nap‐To‐Nap Stability of Slow‐Wave‐Spindle Coupling

For each nap, the phase of slow wave–spindle coupling during N2 sleep was calculated separately for slow and fast spindles. First, the EEG data were filtered between 0.5 and 2 Hz. The instantaneous phase of the slow wave signal was estimated using the Hilbert transform, and phase angles were extracted. Fast and slow spindles occurring within the time window between the negative zero‐crossing (slow wave onset) and the endpoint of each slow wave were identified. For each detected slow wave, the corresponding (fast or slow) spindle peak within this temporal window was extracted. Phase timing was calculated using circular statistics (Circular Statistics Toolbox MATLAB; Berens 2009), including the circular mean and resultant vector length (r). This enabled the evaluation of the preferred phase at which spindles align with slow waves and the strength of their coupling. Nap‐to‐nap stability of the preferred phase of spindle–slow wave coupling was assessed at each electrode using circular correlation (*circ_corrcc.m*).

To further assess the stability of slow wave‐spindle coupling, we performed a complementary phase‐amplitude coupling (PAC) analysis quantifying the modulation of sigma amplitude (slow: 8–13 Hz; fast: 13–18 Hz) by the phase of delta oscillations (0.5–2 Hz). We adopted the same PAC analysis approach as Cross et al. (2025), with the key difference that coupling was evaluated within the time window of each detected slow wave instead of over standard fixed epochs. A detailed description of the methods is provided in the S1. Circular correlations between Nap 1 and Nap 2 preferred coupling phases were used to estimate the nap‐to‐nap stability of the delta‐sigma phase relationship.

## 3 Results

On average, each subject spent on each nap 62.06 ± 15.37 min asleep, of which 57.96 ± 13.11 min were in NREM sleep (N1 = 10.71 ± 5.33 min; N2 = 32.32 ± 10.48 min; N3 = 14.92 ± 11.03 min). Only 9 subjects out of 19 entered REM sleep during their naps (mean: 4.10 ± 5.60 min; also see Figure S1). A general trend toward reduced time spent in all sleep stages was observed in Nap 1 compared to Nap 2 (mean N1 Nap 1: 12.81 min, mean N1 Nap 2: 8.61 min, *p* = 0.055; mean N2 Nap 1: 28.23 min, mean N2 Nap 2: 36.42 min, *p* = 0.078; mean N3 Nap 1: 11.94 min, mean N3 Nap 2: 17.89 min, *p* = 0.148) (see Table 1). Sleep onset latency (SOL, defined as N1 sleep latency) was longer in Nap 1 than in Nap 2 (Nap 1: 11.61 ± 11.95 min; Nap 2: 6.28 ± 7.80 min; W = 131, *p* = 0.047, *r* = 0.48). Similarly, N2 sleep latency was significantly reduced in Nap 2 (Nap 1: 18.39 ± 12.94 min; Nap 2: 10.94 ± 7.92 min; W = 122, *p* = 0.041, *r* = 0.49), and sleep efficiency (SE) was higher in Nap 2 compared to Nap 1 (Nap 1: 60.49% ± 21.47%; Nap 2: 76.34% ± 20.29%; W = 32, *p* = 0.026, *r* = 0.52). This pattern may reflect adaptation difficulties during the first nap session. No other differences in sleep architecture reached significance. Regarding subjective measures, significant differences between the two naps were observed in subjective sleepiness, as measured by the Stanford Sleepiness Scale (Nap 1: 2.73 ± 1.04; Nap 2: 2.00 ± 0.81; *W* = 38, *p* = 0.009, *r* = 0.61). Full test statistics and effect sizes are reported in Table 1. Reported *p* values are not adjusted for multiple comparisons. Applying FDR correction separately to the two logical families of tests (sleep architecture and questionnaires) resulted in all *q*‐values exceeding 0.081, except for the SSS scores, which remained significant at *q* = 0.036.

### 3.1 N2 Sleep Spectral Power Shows Frequency Band–Specific Reliability Across Naps

**FIGURE 1:**
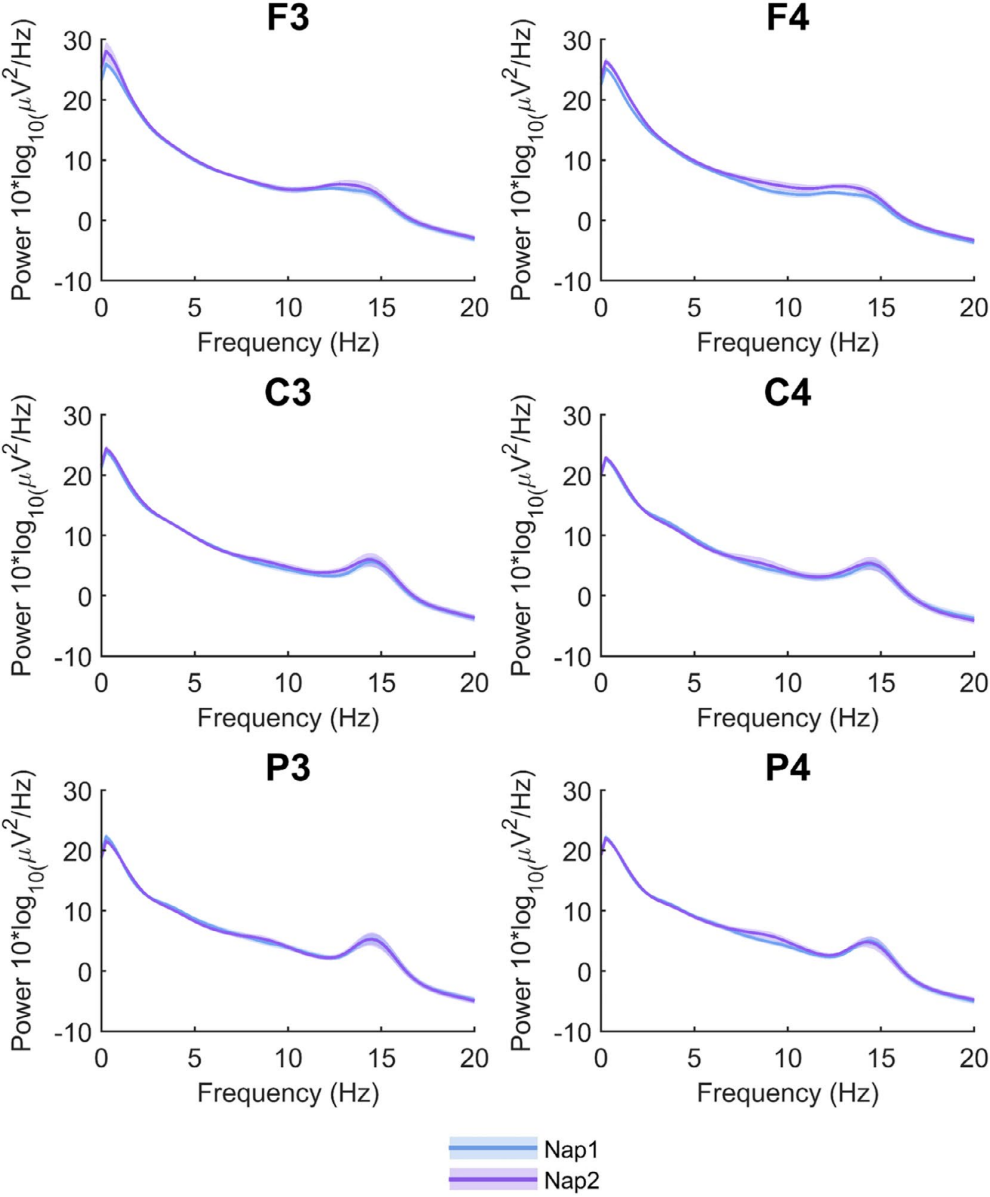
Mean spectral power distributions during N2 sleep for Nap 1 (blue) and Nap 2 (purple). Power spectra are shown for frontal (F3, F4), central (C3, C4), and parietal (P3, P4) electrodes across the 0–20 Hz frequency range. Shaded areas represent the standard error of the mean.

We first examined the spectral power distributions cleaned from artefacts in N2 sleep epochs. Distributions demonstrated highly consistent profiles across the two nap sessions. As shown in Figure 1, the average power spectra across frontal (F3, F4), central (C3, C4), and parietal (P3, P4) electrodes revealed the expected 1/f pattern, with clear peaks in the spindle frequency range (approximately 12–13 Hz in frontal electrodes and 14–15 Hz in centro‐parietal electrodes). Visual inspection indicates overlap between the two sessions for the majority of channels and participants (also see Figure S2).

This qualitative stability was only partially supported by the intraclass correlation coefficients (ICCs) computed for each electrode's broadband power values, which indicated generally moderate test–retest reliability across N2 nap sleep: F3: ICC = 0, (95% CI [0 0.46]), *p* = 0.604; Fz: ICC = 0.44, (95% CI [0 0.76]), *p* = 0.045; F4: ICC = 0.50, (95% CI [0 0.79]), *p* = 0.013; C3: ICC = 0.45, (95% CI [0 0.79]), *p* = 0.046; C4: ICC = 0.60 (95% CI [0 0.86]), *p* = 0.018; P3: ICC = 0.66 (95% CI [0.19 0.87]), *p* = 0.005; P4: ICC = 0.76 (95% CI [0.43 0.91]), *p* < 0.001. However, after controlling for multiple comparisons using the FDR correction, only the results for P3 and P4 remained statistically significant (*q* < 0.05). We also computed the intraclass coefficients (ICCs) for delta (0.5–4 Hz) and alpha/sigma power, both in the slow (8–13 Hz) and fast band (13–18 Hz). The ICCs for delta power showed moderate nap‐to‐nap stability (ICC range 0.45–0.74 in fronto‐centro‐parietal electrodes). Following FDR correction, only the results in F3, P3, and P4 remained significant (qs < 0.01). In contrast, both low and high sigma power showed optimal nap‐to‐nap stability across all examined electrodes (low: ICC range 0.62–0.88, all ps < 0.01; high: ICC range 0.81–0.92, all ps < 0.001). All findings on sigma power remained statistically significant after controlling for multiple comparisons (all qs < 0.01).

### 3.2 Characteristics of Sleep Spindles Are Stable Across Naps

Sleep spindle characteristics—including peak frequency, duration, and density—were systematically analysed across both nap sessions. These analyses encompassed all spindles, as well as the fast and slow spindle subtypes (see Tables S3 and S4 for the number of detected events for each subtype). Subject‐level means for each metric were extracted from seven key electrodes (F3, Fz, F4, C3, C4, P3, and P4), representing frontal and centro‐parietal regions where sleep spindles are most evident in EEG recordings (Andrillon et al. 2011).

**FIGURE 2:**
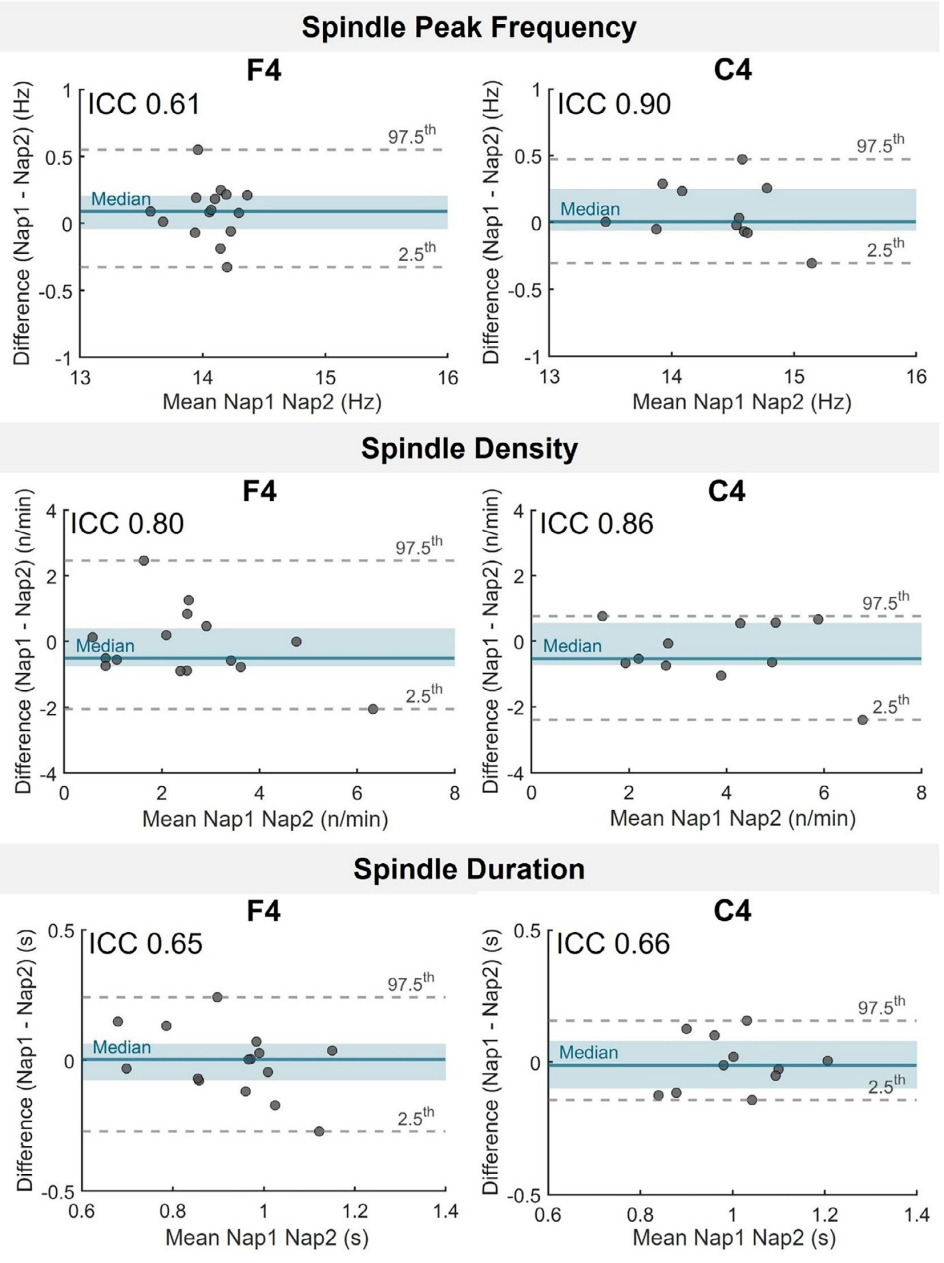
Between‐nap reliability of fast sleep spindle properties. Bland–Altman plots show the participant‐level differences between Nap 1 and Nap 2 sessions for three sleep spindles proprieties (Peak Frequency, Duration, and Density) at F4 and C4 electrodes. Each dot represents one participant. Teal blue lines show the median difference; shaded areas mark the interquartile range (IQR). Grey dashed lines indicate the 2.5th and 97.5th percentiles. ICC values indicate significant reliability after FDR correction (q < 0.05).

**FIGURE 3:**
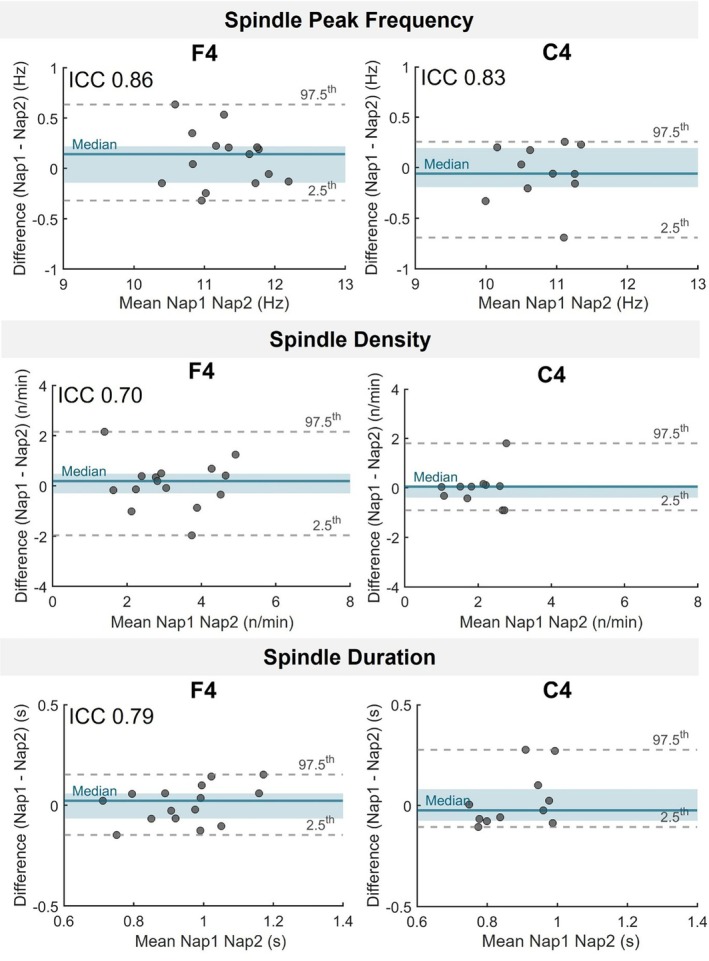
Between‐nap reliability of slow sleep spindle properties. Bland–Altman plots show the participant‐level differences between Nap 1 and Nap 2 sessions for three sleep spindles proprieties (Peak Frequency, Duration, and Density) at F4 and C4 electrodes. Each dot represents one participant. Teal blue lines show the median difference; shaded areas mark the interquartile range (IQR). Grey dashed lines indicate the 2.5th and 97.5th percentiles. ICC values indicate significant reliability after FDR correction (*q* < 0.05).

Peak frequency of sleep spindles demonstrated moderate to excellent reliability across nearly all electrodes, especially in frontal and central regions, regardless of spindle subtype (see Table 2, Figures 2, and 3). For illustrative purposes, Figures 2 and 3 will display data from a representative subset of two electrodes (F4 and C4) for slow and fast spindles, respectively.

**TABLE 2:** Mean reliability indices (ICC) for sleep spindles measures across electrodes.

	F3				Fz				F4				C3				C4				P3				P4			
	ICC	LB	UB	*p*	ICC	LB	UB	*p*	ICC	LB	UB	*p*	ICC	LB	UB	*p*	ICC	LB	UB	*p*	ICC	LB	UB	*p*	ICC	LB	UB	*p*
Peak frequency	0.97	0.90	0.99	< 0,001	0.94	0.83	0.98	< 0,001	0.93	0.82	0.98	< 0,001	0.86	0.57	0.96	< 0,001	0.77	0.35	0.93	0.002	0.77	0.37	0.93	0.001	0.63	0.22	0.86	0.003
Peak frequency fast	0.74	0.37	0.91	< 0,001	0.66	0.25	0.87	0.002	0.61	0.18	0.84	0.004	0.82	0.50	0.94	< 0,001	0.90	0.69	0.97	< 0,001	0.88	0.65	0.96	< 0,001	0.88	0.68	0.96	< 0,001
Peak frequency slow	0.89	0.69	0.97	< 0,001	0.84	0.58	0.94	< 0,001	0.86	0.65	0.95	< 0,001	0.86	0.58	0.96	< 0,001	0.83	0.50	0.95	< 0,001	0.79	0.45	0.94	< 0,001	0.50	0.05	0.79	0.016
Density	0.57	0.09	0.84	0.012	0.63	0.19	0.86	0.004	0.54	0.03	0.82	0.019	0.82	0.50	0.95	< 0,001	0.92	0.74	0.98	< 0,001	0.07	−0.58	0.61	0.42	0.71	0.32	0.89	0.001
Density fast	0.89	0.70	0.97	< 0,001	0.89	0.70	0.96	< 0,001	0.80	0.49	0.93	< 0,001	0.87	0.60	0.96	< 0,001	0.86	0.58	0.96	< 0,001	0.47	−0.15	0.81	0.063	0.76	0.42	0.91	< 0,001
Density slow	0.63	0.17	0.87	0.006	0.68	0.28	0.88	0.002	0.70	0.30	0.89	0.002	0.63	0.10	0.88	0.013	0.55	−0.08	0.86	0.040	0.24	−0.42	0.71	0.227	0.29	−0.26	0.69	0.145
Duration	0.68	0.24	0.89	0.003	0.78	0.47	0.92	< 0,001	0.75	0.40	0.91	< 0,001	0.74	0.33	0.92	0.001	0.60	0.00	0.87	0.026	0.69	0.22	0.90	0.005	0.70	0.30	0.89	0.002
Duration fast	0.76	0.39	0.92	< 0,001	0.72	0.34	0.90	0.001	0.65	0.21	0.87	0.004	0.78	0.41	0.93	0.001	0.66	0.11	0.90	0.013	0.32	−0.32	0.75	0.152	0.72	0.33	0.90	0.001
Duration slow	0.61	0.15	0.86	0.006	0.63	0.18	0.86	0.005	0.79	0.48	0.93	< 0,001	0.39	−0.25	0.78	0.105	0.34	−0.33	0.77	0.149	0.37	−0.21	0.77	0.099	0.27	−0.31	0.68	0.173

*Note:* This table presents the intra‐class correlation coefficients (ICCs), along with their 95% confidence intervals (lower bound—LB, upper bound—UB) and corresponding *p* values, for various sleep spindle parameters recorded at scalp electrodes (F3, Fz, F4, C3, C4, P3, and P4). Measures are reported separately for all spindles, as well as for fast and slow spindles. The evaluated parameters include Peak Frequency (Hz), Density (n/min), and Duration (s). Grey shading indicates ICCs that were significant after FDR correction (q < 0.05).

Spindle density and duration also showed significant stability in frontal and central sites, but their reliability was generally lower and not significant at parietal electrodes, particularly for slow spindles. Fast spindle properties showed the greatest consistency across naps, making them the most reliable measures for assessing individual sleep differences over repeated nap sessions. All statistical results are presented in Table 2.

### 3.3 Slow Wave Characteristics Show Moderate Nap‐To‐Nap Stability

**FIGURE 4:**
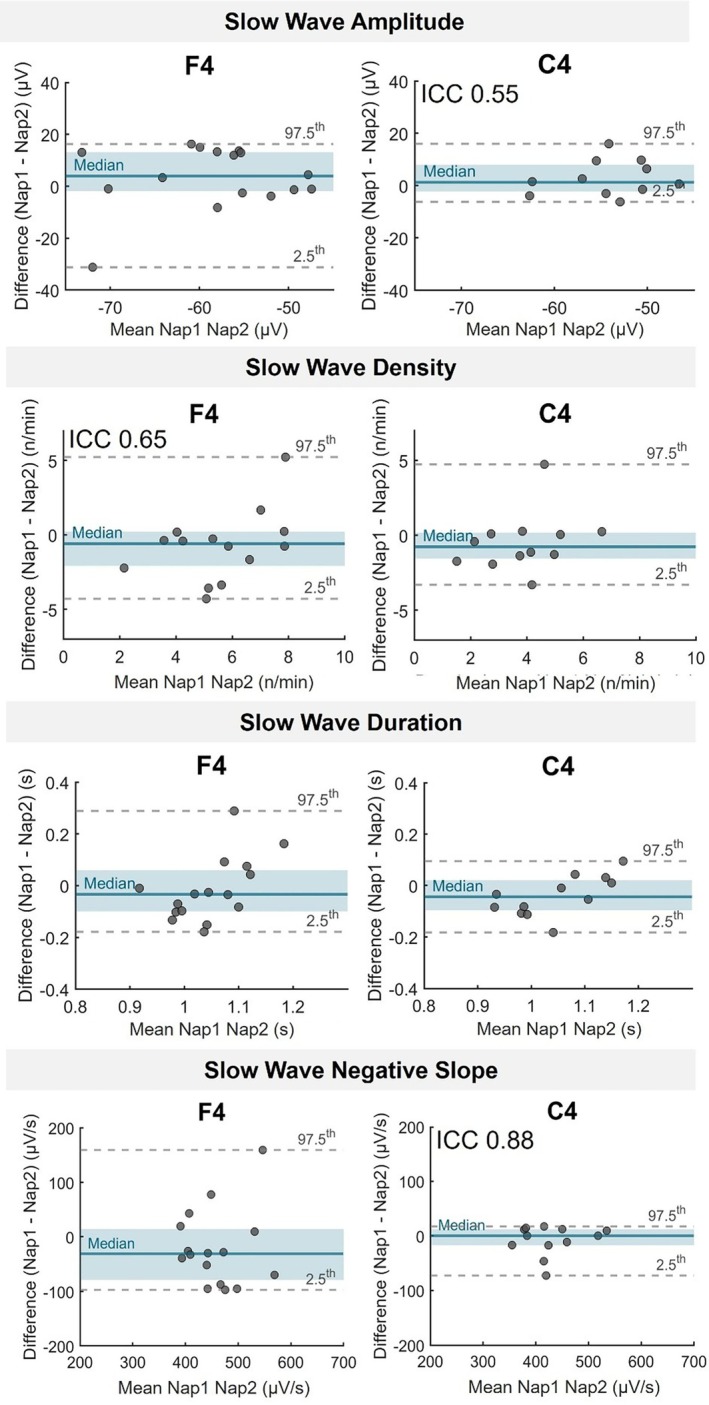
Between‐nap reliability of slow wave properties. Bland–Altman plots show the participant‐level differences between Nap 1 and Nap 2 sessions for four slow‐wave features (Amplitude, Duration, Density, and Down Slope) at electrodes F4 and C4. Each dot represents the difference value of a single participant. Teal blue lines show the median difference; shaded areas mark the interquartile range (IQR). Grey dashed lines indicate the 2.5th and 97.5th percentiles. ICC values indicate significant reliability after FDR correction (*q* < 0.05).

The characteristics of detected slow waves were analyzed across both naps, focusing on amplitude, duration, density, and negative slope (Figure 4). For illustrative purposes, Figure 4 will display data from a representative subset of two electrodes (F4 and C4). Subject means for each metric were assessed using data from five electrodes (F3, Fz, F4, C3, and C4) selected to represent the frontal and central regions, which most prominently display slow wave activity (Murphy et al. 2009).

As shown in Table 3, slow wave density exhibited the highest nap‐to‐nap reliability among all metrics, with significant ICCs observed across both frontal and central electrode sites. Similarly, slow wave amplitude exhibited moderate stability in Fz and C4. The negative slope of slow waves was also stable across naps, in frontal and central derivations. Slow wave duration demonstrates no reliability across naps.

**TABLE 3:** Mean reliability indices (ICC) for slow wave measures across electrodes.

	F3				Fz				F4				C3				C4			
	ICC	LB	UB	*p*	ICC	LB	UB	*p*	ICC	LB	UB	*p*	ICC	LB	UB	*p*	ICC	LB	UB	*p*
Amplitude	−0.14	−0.66	0.43	0.678	0.53	0.05	0.81	0.017	0.27	−0.23	0.66	0.145	0.43	−0.15	0.78	0.068	0.55	0.05	0.84	0.017
Density	0.60	0.12	0.85	0.010	0.60	0.14	0.85	0.008	0.65	0.22	0.87	0.003	0.61	0.14	0.86	0.008	0.36	−0.30	0.78	0.133
Duration	0.08	−0.47	0.58	0.388	0.44	−0.08	0.77	0.047	0.09	−0.46	0.57	0.375	0.07	−0.51	0.59	0.409	0.57	0.04	0.86	0.018
Negative slope	0.03	−0.53	0.54	0.465	0.61	0.17	0.85	0.005	0.40	−0.08	0.74	0.050	0.65	0.18	0.88	0.006	0.88	0.63	0.97	< 0,001

*Note:* This table shows intra‐class correlation coefficients (ICC), 95% confidence intervals (lower bound—LB, upper bound—UB), and corresponding *p* values for slow wave parameters measured at front‐central scalp electrodes (F3, Fz, F4, C3, and C4). Parameters include Amplitude (μV), Density (n/min), Duration (s), and Negative Slope (μV/s). Grey shading indicates ICCs that were significant after FDR correction (*q* < 0.05).

### 3.4 Inconsistent Nap‐To‐Nap Stability of Spindle‐Slow Wave Phase Coupling

To further examine spindle‐slow wave phase coupling, we assessed the consistency of their specific temporal alignment across naps. The EEG signal was bandpass filtered, and the instantaneous phase was extracted via the Hilbert transform. The slow wave phase at each spindle peak was then determined.

In the first nap, the mean density of N2 slow spindles coupled with slow waves was 1.15 ± 0.63 events per minute at the F4 electrode and 0.58 ± 0.39 at C4. In the second nap, these values were 1.19 ± 0.53 at F4 and 0.65 ± 0.31 at C4. In contrast, the mean density of N2 fast spindles coupled with slow waves was 0.63 ± 0.34 events per minute at F4 and 0.64 ± 0.30 at C4 in the first nap, increasing to 0.76 ± 0.44 at F4 and 0.91 ± 0.42 at C4 in the second nap.

**FIGURE 5:**
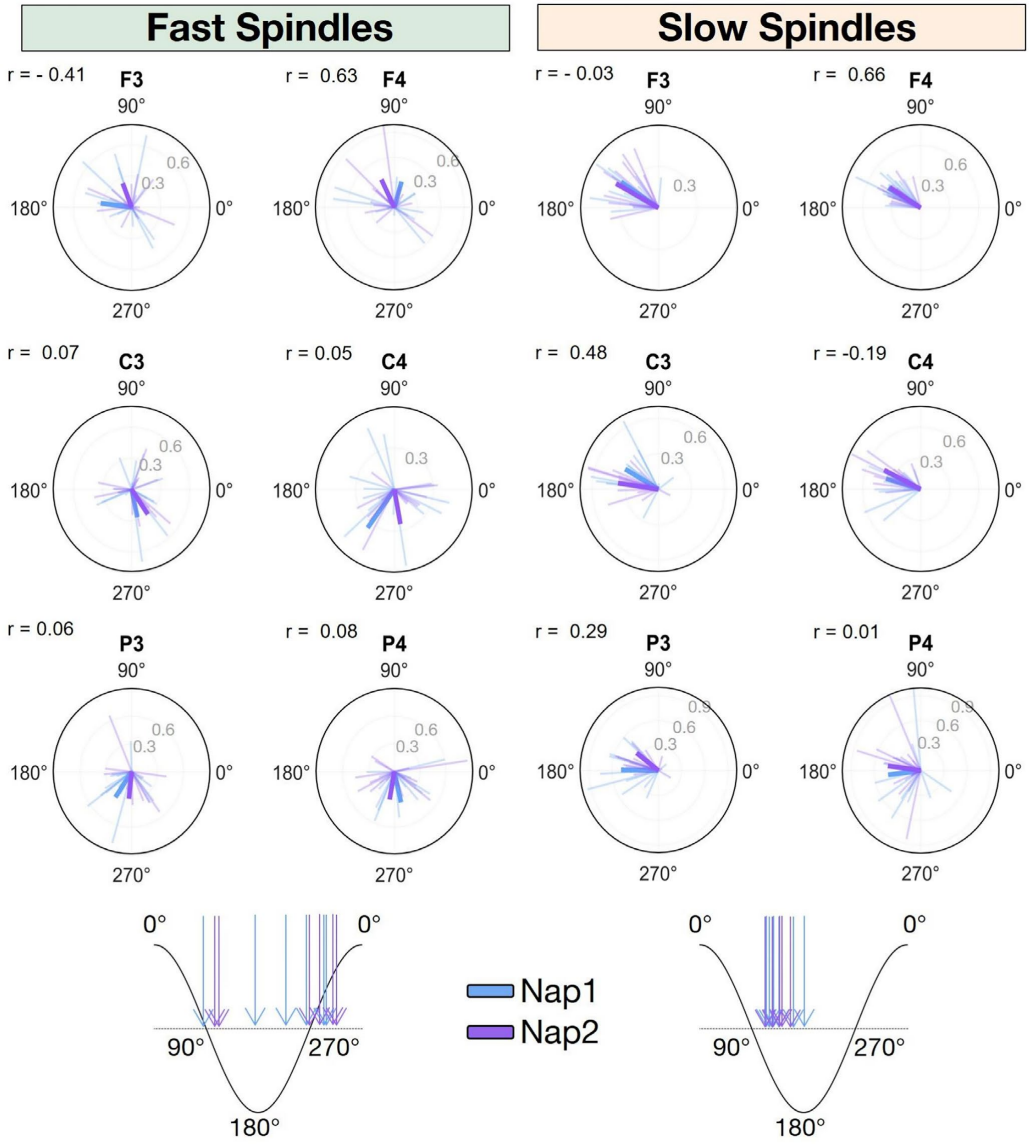
Circular plots showing the phase coupling between slow waves and spindles during N2 sleep for fast spindles (left panel) and slow spindles (right panel) across two nap sessions (Nap 1 in blue, Nap 2 in purple). For each EEG channel, the phase of the slow wave at the spindle peak was computed separately for fast and slow spindles, considering only events occurring between the negative zero‐crossing and the endpoint of each slow wave. Vectors represent the circular mean direction and the strength of phase locking (resultant vector length) for each participant (shaded colours) and for participants' mean (dark colours). r: Circular correlation coefficient.

As shown in the circular plots in Figure 5, fast spindles predominantly occurred during the depolarizing (up) phase of the slow wave, although with substantial topographic inter‐nap variability. In contrast, slow spindles tended to occur during the hyperpolarized (down) phase of the slow wave, exhibiting lower nap‐to‐nap variability in fronto‐central electrodes (also see Figure S3). Despite these general patterns, the preferred phase of spindle‐slow wave coupling varied markedly across sessions and participants. Correlation analyses revealed no significant relationship between Nap 1 and Nap 2 phase angles across channels and spindle type (all *q*‐values > 0.05 after FDR correction).

To complement and validate the event‐based findings, we performed a phase–amplitude coupling (PAC) analysis, quantifying the modulation of sigma amplitude (slow: 8–13 Hz; fast: 13–18 Hz) by the phase of delta oscillations (0.5–2 Hz). This approach allowed us to overcome the methodological limitations inherent to spindle event detection, which can be sensitive to threshold choices and inter‐individual variability. Consistent with the event‐based results, no significant correlation of preferred delta‐sigma phases was observed between Nap 1 and Nap 2 across electrodes (all *q* > 0.05, FDR‐corrected; Figure S4).

These findings suggest that the precise timing of spindle occurrence relative to the slow wave phase is not consistently maintained across naps.

## 4 Discussion

In the present exploratory study, we investigated the nap‐to‐nap reliability of electrophysiological features of NREM sleep in healthy young adults. Overall, our findings demonstrate a high level of intra‐individual consistency in the morphological characteristics of sleep spindles (e.g., peak frequency, duration, and density). This finding has also been confirmed by assessing the spectral power in the sigma range, which showed the greatest reliability compared to both delta and broadband power values. Slow wave properties (amplitude, slope, duration, and density) demonstrated moderate to absent stability. Instead, the timing of spindle‐slow wave coupling was more variable across subjects and did not show consistent reliability between naps.

This general pattern aligns with previous evidence on overnight NREM power stability: quantitative NREM spectra are stable across nights, as they appear to be genetically determined (Buckelmüller et al. 2006; de Gennaro et al. 2008), especially when considering the sigma range (De Gennaro et al. 2005).

The trait‐like stability extends to sleep spindle characteristics, which are highly consistent across nights and subjects (Cox et al. 2017; Cross et al. 2025; Eggert et al. 2015; Mullins et al. 2025; Reynolds et al. 2019). For instance, Purcell et al. (2017) found that overnight spindle parameters in a cohort of over 4000 individuals were consistent after more than 5 years and strongly influenced by genetics (Purcell et al. 2017).

Our data also reflect slight differences between fast and slow spindles: fast spindle metrics were generally more reliable than slow spindle metrics in all considered channels, consistent with findings by Reynolds et al. (2019). In their multi‐night study on adolescents, fast spindle density, duration, and amplitude achieved acceptable reliability on a single recording, whereas slow spindle density and duration required aggregation over several nights. The observed difference may stem from both neurophysiological and methodological considerations. Neurophysiologically, fast spindles originate from stable thalamo‐cortical core circuits, while slow spindles may rely on less consistent mechanisms, including non‐specific thalamic projections and local cortical generators, resulting in greater variability (Ayoub et al. 2013; Staresina et al. 2015; Timofeev and Chauvette 2013). Methodologically, fixed‐band detection algorithms reliably capture fast spindles but appear to be less effective for slow spindles, whose properties vary more across individuals (Ujma et al. 2015). A third, yet unresolved hypothesis, closely related to the first, posits that fast and slow spindles may subserve partially distinct functions throughout sleep (Mölle et al. 2011; Ng et al. 2024). While both types are implicated in memory consolidation and synaptic plasticity, fast spindles may reflect more deterministic hippocampal‐thalamo‐cortical dynamics (Staresina et al. 2015), whereas slow spindles could be indicative of higher‐order cortical processes, including local learning effects or fluctuating sleep pressure that drive greater inter‐session variability.

Moreover, our results indicated that the reliability of slow wave activity and the morphological features of slow waves across naps was generally moderate to low, or in some cases, absent. This lower stability relative to sleep spindle properties may be attributed to the well‐documented sensitivity of slow waves to state‐dependent factors, such as sleep pressure, prior wake duration (Borberly and Achermann 1999), or recent learning experiences (Bernardi et al. 2019; Huber et al. 2004). Indeed, although naps are globally less affected by cumulative homeostatic and circadian influences compared to nocturnal sleep, the first NREM cycle of a daytime nap may still reflect local, short‐term homeostatic modulation, particularly in the brain regions where slow waves are expressed (Riedner et al. 2007).

A similar explanation may underlie the absence of reliable slow‐wave‐spindle coupling observed in our study. Although we mostly replicated the overnight finding that fast spindles align with the depolarizing (up‐state) phase of the slow wave and slow spindles with the hyperpolarizing phase (Klinzing et al. 2016; Staresina et al. 2015; also see Supporting Information: Results), our data from individual nap sessions revealed no stable coupling between spindle and slow‐wave events. This is surprising, as previous overnight investigations found that spectral metrics of slow‐wave and spindle coupling are remarkably stable across nights, functioning almost like an electrophysiological fingerprint (Cox et al. 2018; Cross et al. 2025). Those studies, however, relied on phase‐amplitude coupling analysis rather than discrete event detection. In the present work, we additionally performed a PAC analysis focusing on the phase of delta activity and the amplitude of the sigma band, separately for fast and slow sigma frequencies. Unlike previous studies that computed PAC over fixed time windows, we evaluated coupling within the temporal boundaries of each detected slow wave. Consistent with the event‐based results, these analyses revealed no significant nap‐to‐nap correlation in the preferred phase of coupling across electrodes. Thus, both analytical approaches converge in showing that spindle–slow‐wave coupling during daytime naps lacks the robust stability previously observed during overnight sleep. Given our limited number of spindle‐slow wave occurrences, additional research, ideally involving larger datasets, is needed to determine whether this phase inconsistency is attributable to inherent physiological variability, to differences between nap and nocturnal sleep, or to low statistical power. Still, rather than representing a stable individual sleep fingerprint, we propose that spindle‐slow wave coupling timing reflects a variable process modulated by transient region‐specific fluctuations in cortical excitability (Bernardi et al. 2019) and ongoing memory consolidation demands (Kam et al. 2019; Niknazar et al. 2015). Indeed, the precise temporal and spatial coupling of sleep spindles to the peak of slow waves coincides with the transient occurrence of hippocampal sharp‐wave ripples, which are thought to support memory consolidation (Muehlroth et al. 2019; Staresina et al. 2023). This coordination is particularly pronounced for fast spindles, which show the strongest temporal association with hippocampal ripples (Staresina et al. 2015). The transient nature of these coordinated events suggests their coupling patterns adapt to support specific memory processing needs. The observed topographic heterogeneity of the phase coupling between slow waves and fast spindles likely reflects both fewer detected events (and thus noisier phase estimates), especially over frontal channels, and regional differences in the timing of hippocampo‐thalamo‐cortical interactions modulating fast spindle generation (Muehlroth et al. 2019; Staresina et al. 2015).

The findings of this exploratory study should be interpreted in light of several limitations. First, the sample size was relatively small and consisted exclusively of healthy young adults, which limits the generalizability of the results. Second, topographical analyses were not performed due to the limited number of EEG electrodes and the exclusion of several channels due to poor signal. The removal of these channels may have further reduced the precision of ICC estimates for certain metrics. Although this represents a limitation, it also underscores the feasibility of investigating sleep microstructure using a reduced EEG montage, which is commonly adopted in clinical settings. Another important limitation concerns the relatively high attrition rate (∼41%), which substantially reduced the final sample size. This is not uncommon in nap studies conducted in controlled laboratory environments, where some participants may find it challenging to initiate and maintain sleep. Notably, difficulties in initiating sleep (as reflected in SOL and N2 sleep latency) and in reaching good sleep efficiency appeared more pronounced during the first session, reflecting an adaptation effect to the laboratory environment. Importantly, only individuals who were able to sleep in both nap sessions were retained, which may have introduced a degree of selection bias. Moreover, pre‐nap state was not systematically controlled beyond the use of subjective questionnaires. In particular, no actigraphy or sleep diaries were employed to monitor sleep–wake patterns, wake‐up times, or the consumption of caffeine and other stimulants. These uncontrolled factors may have influenced homeostatic sleep pressure and subjective sleepiness, thereby contributing to intra‐individual variability across sessions. Of note, subjective sleepiness was significantly higher before the first nap compared to the second, which may reflect differences in homeostatic sleep pressure or variability in pre‐nap cognitive and physical activity between sessions. In general, adopting strategies to minimise attrition and variability, such as partial sleep restriction prior to the nap, inclusion of an adaptation nap, and closer monitoring of the pre‐nap state, should be considered in future research.

To confirm our results, future studies should include larger samples, employ high‐density EEG caps, adopt more standardised protocols, control for potential confounding variables, and implement different event detection methods. Moreover, additional longitudinal investigations are required to better characterise the individual stability of overnight or nap sleep graphoelements throughout the lifespan.

To conclude, our results suggest that many basic electrophysiological features of NREM sleep, particularly sleep spindles, are relatively stable, trait‐like markers that may serve as individual fingerprints of sleep, even during naps. In contrast, more complex dynamics, such as the timing of slow wave–spindle coupling, appear to vary substantially across nap sessions. Given that nap NREM sleep closely resembles the characteristics of nocturnal NREM sleep, daytime recordings could represent a practical alternative to full‐night studies (Mylonas et al. 2020). This approach may facilitate faster and more practical sleep assessments across research and clinical contexts.

## Author Contributions

Conceptualization: N.C. Investigation: D.B. and A.V. Methodology: N.C. and A.V. Software: N.C., A.V., and D.B. Formal analysis: A.V., D.B., and A.B. Visualisation: D.B., A.V., and A.B. Supervision: N.C. Funding acquisition: N.C. Project administration: N.C. Data curation: D.B. and A.V. Writing – original draft: D.B. Writing – review and editing: All authors. All authors have read and agreed to the published version of the manuscript.

## Conflicts of Interest

The authors declare no conflicts of interest.

## Supporting information


**Figure S1:** jsr70253‐sup‐0001‐supinfo.docx. Sleep architecture and event detection.
**Figure** **S1**. (a) Amount of total time spent in sleep and wake stage for each participant, summed across the two nap sessions. Each column represents a different subject. (b) Amount of sleep time spent in each stage for each participant, divided by nap session. c) Example of a 30‐s EEG segment from the F4 channel, showing detected sleep spindles (in pink) and slow waves (in light blue).
**Table S1:**. Number of detected slow waves.
**Table S1:**. NaN indicates removed channels.
**Table S2:**. Number of detected sleep spindles.
**Table S2:**. NaN indicates removed channels.
**Table S3:**. Number of detected fast sleep spindles.
**Table S3:**. NaN indicates removed channels.
**Table S4:**. Number of detected slow sleep spindles.
**Table S4:**. NaN indicates removed channels.
**Figure S2:**. NREM2 sleep EEG power spectra by participant and session.
**Figure S2:** Power spectra are displayed for frontal (F3: dark blue, F4: orange), central (C3: yellow, C4: purple), and parietal (P3: green, P4: light blue) electrodes across the 0–20 Hz frequency range. For each participant, only electrodes retained for analysis are displayed.
**Figure S3:** Spindle‐slow wave coupling mean phase differences (Nap1–Nap2).
**Figure S3:** Circular plots showing the mean difference in phase coupling of spindles to slow waves between two nap sessions (Nap 1–Nap 2) during N2 sleep, displayed separately for fast spindles (left panel) and slow spindles (right panel). Each plot corresponds to a specific EEG electrode, and each line represents an individual participant, colour‐coded consistently across plots. A direction of 0° indicates no change in preferred coupling phase between Nap 1 and Nap 2, whereas angles distant from 0° reflect a shift in coupling phase across sessions.
**Figure S4:** Preferred delta phase (0.5–2 Hz) modulating sigma amplitude (8–13 Hz and 13–18 Hz) within slow wave events across two nap sessions.
**Figure S4:** Circular plots showing the preferred delta phase (0.5–2 Hz) associated with maximal sigma amplitude (fast: 13–18 Hz and slow: 8–13 Hz) within the detected slow wave events across two nap sessions (Nap 1 in blue, Nap 2 in purple). For each EEG channel, the coupling between delta phase and sigma amplitude was computed separately for fast and slow sigma, considering only the signal between the negative zero‐crossing and the endpoint of each slow wave. Vectors represent the circular mean direction and the strength of phase locking (resultant vector length, (r) for each participant (shaded colours) and for participants' mean (dark colours). r: circular correlation coefficient.
**Figure S5:** Temporal dynamics of spindle‐slow wave co‐occurrence in naps.
**Figure S5: (**a) Time‐frequency plots of event‐related spectral perturbation (ERSP) for frontal (F3, F4), central (C3, C4), and parietal (P3, P4) electrodes, combining data from Nap 1 and Nap 2. Time 0 represents the event onset—slow wave negative peak. The black waveform superimposed on each plot represents the average slow wave. ERSP plots show power modulations in decibels (dB). (b) Event correlation histograms (ECHs) of sleep spindles around slow wave negative peaks. The histograms illustrate the rate of detected spindle occurrence (events/s) within a ± 3‐s window centered on the negative peak of the slow wave, with bins of approximately 63 ms. Dark violet bars indicate the mean ECH values with their standard error, while pink bars represent the mean of randomised ECHs (rECHs) obtained by jittering the timing of slow wave peaks within ±3 s, each with corresponding standard error bars. Asterisks above the bars indicate bins with significant differences between observed and randomised data (*p* < 0.05), determined through paired‐sample t‐tests (5000 permutations) and corrected for multiple comparisons using Threshold‐Free Cluster Enhancement (TFCE). A black dotted line shows the normalised mean slow wave amplitude for each channel, serving as a temporal reference for spindle occurrences. Data from only six representative electrodes (F3, F4, C3, C4, P3, and P4) are displayed.

## Data Availability

The data that support the findings of this study are openly available in OSF at https://osf.io/z5r2j/.
