# Supplementary material for: Differential Nap‐To‐Nap Stability of Sleep Spindles, Slow Waves, and their Temporal Coupling: An Exploratory Study
Source: J Sleep Res. 2025 Nov 25;35(3):e70253. doi: 10.1111/jsr.70253 (PMC13193487; doi:10.1111/jsr.70253)
Supplement: Supplementary file 1 — Figure S1: jsr70253‐sup‐0001‐supinfo.docx. Sleep architecture and event detection. Figure S1 . (a) Amount of total time spent in sleep and wake stage for each participant, summed across the two nap sessions. Each column represents a different subject. (b) Amount of sleep time spent in each stage for each participant, divided by nap session. c) Example of a 30‐s EEG segment from the F4 channel, showing detected sleep spindles (in pink) and slow waves (in light blue). Table S1:. Number of detected slow waves. Table S1:. NaN indicates removed channels. Table S2:. Number of detected sleep spindles. Table S2:. NaN indicates removed channels. Table S3:. Number of detected fast sleep spindles. Table S3:. NaN indicates removed channels. Table S4:. Number of detected slow sleep spindles. Table S4:. NaN indicates removed channels. Figure S2:. NREM2 sleep EEG power spectra by participant and session. Figure S2: Power spectra are displayed for frontal (F3: dark blue, F4: orange), central (C3: yellow, C4: purple), and parietal (P3: green, P4: light blue) electrodes across the 0–20 Hz frequency range. For each participant, only electrodes retained for analysis are displayed. Figure S3: Spindle‐slow wave coupling mean phase differences (Nap1–Nap2). Figure S3: Circular plots showing the mean difference in phase coupling of spindles to slow waves between two nap sessions (Nap 1–Nap 2) during N2 sleep, displayed separately for fast spindles (left panel) and slow spindles (right panel). Each plot corresponds to a specific EEG electrode, and each line represents an individual participant, colour‐coded consistently across plots. A direction of 0° indicates no change in preferred coupling phase between Nap 1 and Nap 2, whereas angles distant from 0° reflect a shift in coupling phase across sessions. Figure S4: Preferred delta phase (0.5–2 Hz) modulating sigma amplitude (8–13 Hz and 13–18 Hz) within slow wave events across two nap sessions. Figure S4: Circular plots showing the preferred [file JSR-35-e70253-s001.docx]

**Supplementary material**

**Differential nap-to-nap stability of sleep spindles, slow waves, and their temporal coupling: an exploratory study**

Damiana Bergamo ^1^, Antonino Visalli ^1,2^, Angie Baldassarri ^1^, Nicola Cellini ^1^

## **SUPPLEMENTARY METHODS**

## **Time frequency decomposition**

We used EEGLAB’s *newtimef* function to estimate event-related spectral perturbation (ERSP) around detected slow wave events. To explore power changes across all slow wave events, data from the two naps were merged, filtered between 0.16 and 40 Hz, and segmented into epochs of ±3 s around each slow wave’s negative peak. Time-frequency decomposition was performed using 150 linearly spaced frequencies from 6 to 20 Hz, applying wavelets whose number of cycles increased linearly (starting at 3 cycles and increasing by 0.5 cycles per Hz). Baseline correction was applied on a trial-by-trial basis, using the average power from 1.8 to 1.5 s before the negative peak of the slow wave as our baseline reference.

## **Temporal co-occurrence of spindles and slow waves**

Event correlation histograms (ECHs) were employed to assess a possible preferential occurrence of spindles within the slow wave time window. For this analysis, data from the two daytime naps were merged. ECHs were calculated using a ±3s window around the negative peaks of slow waves with a bin size of 62.5 ms. Counts of spindle events in each bin were divided by the number of slow waves. Counts were then converted to rates (events/s) to make values independent of bin size.

To statistically evaluate the spindle-slow wave relationship, random ECHs (rECHs) were generated by introducing a pseudo-random temporal jitter within ±3 seconds of the slow wave negative peak. The ept-TFCE (Threshold-Free Cluster-Enhancement) toolbox [(Mensen & Khatami, 2013)](https://www.zotero.org/google-docs/?vsI1pw) was used to conduct a paired-sample *t*-test (number of permutations = 5000, E = 0.66, H = 2, alpha = .05) between ECHs and rECHS.

## **PAC analysis**

For each detected slow wave negative peak, the EEG signal was extracted, including a 2-second buffer on either side of the event to prevent filter edge artifacts. The extracted signal was then bandpass filtered in the delta range (0.5-2 Hz), and the instantaneous phase was obtained as the angle of the Hilbert transform. Simultaneously, the signal was filtered in the corresponding sigma band (slow: 8-13 Hz, fast: 13-18 Hz), and the instantaneous amplitude was calculated as the modulus of the Hilbert transform. Filtering was performed using FIR filters to preserve linear phase properties. After removing the buffer periods, amplitude values were binned according to the corresponding phase values (18 bins), producing a phase-amplitude distribution for each slow wave event (from the first zero crossing to the wave end). Amplitude values were z-scored across bins within each event to reduce variability across events. For each slow wave, the phase bin corresponding to the maximal sigma amplitude was identified. The preferred coupling phase was then computed as the circular mean across all NREM slow-wave events for each electrode and each nap session. To assess the nap-to-nap stability of the preferred delta phase-sigma amplitude relationship, we computed circular correlations across corresponding electrodes.

## **SUPPLEMENTARY RESULTS**

## **Temporal patterns of sigma/spindle and delta/slow wave coupling in daytime naps parallel those observed in nocturnal sleep**

Before assessing the stability of slow wave-spindle co-occurrence, we examined time-frequency dynamics around the negative peak of N2 slow waves across Nap 1 and Nap 2, to evaluate their similarity with patterns reported during nocturnal sleep. In the event-related spectral perturbation (ERSP) results, a prominent increase in power is evident in the theta and low-sigma range shortly before the negative slow wave peak occurrence across all electrode sites, with frontal derivations showing the strongest modulation. In contrast, centro-parietal electrodes (particularly C3, C4, and P3) reveal a high-sigma band (~15 Hz) power increase, which peaks during the up-phase of the slow wave (see Figure S5a).

Since sigma power may encompass noise unrelated to the physiological events of interest, we further investigated the temporal relationship between slow wave dynamics and algorithm-detected spindle events. To this end, we created event correlation histograms (ECHs) by aligning the peak of each spindle to the negative peak of the corresponding slow wave. For this analysis, EEG data from both daytime naps were aggregated. As shown in Supplementary Figure S5b, ECHs revealed an increased spindle occurrence preceding the negative peak of slow waves at frontal electrode sites. In contrast, centro-parietal electrodes showed increased spindle density immediately following the negative peak, likely reflecting fast spindles.

To assess the statistical significance of spindle–slow wave co-occurrence, we generated randomized event-related correlation histograms (rECHs) by temporally jittering spindle events within the same analysis window. A paired-sample t-test with Threshold-Free Cluster Enhancement (TFCE) correction revealed significant temporal clusters of spindle-slow wave coupling in specific EEG channels. In F3, a significant increase in spindle events occurred approximately -200 to -100 ms before the negative peak of the slow wave (all ps < .01); in Fz, from -200 to 100 ms around the negative peak (all ps < .05); in F4, from -300 to 0 ms (all ps < .05); and in C3, from 350 to 450 ms after the negative peak (all ps < .05). Results demonstrate region-specific dynamics of slow wave-spindle interactions during naps, mirroring those observed during nocturnal sleep.

**SUPPLEMENTARY FIGURES AND TABLES**

**Figure S1 | Sleep architecture and event detection**

**
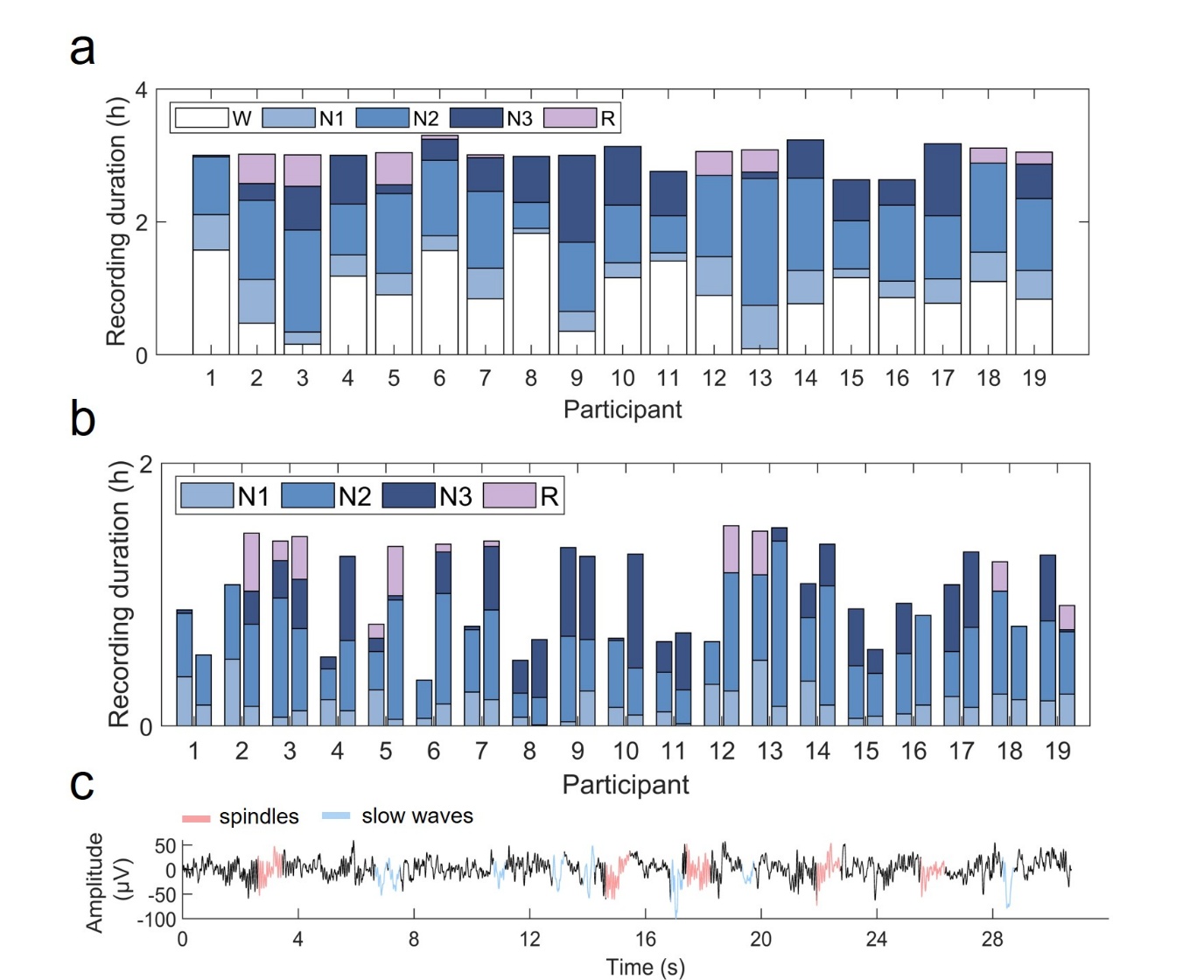
**

**Figure S1 |** a) Amount of total time spent in sleep and wake stage for each participant, summed across the two nap sessions. Each column represents a different subject. b) Amount of sleep time spent in each stage for each participant, divided by nap session. c) Example of a 30-second EEG segment from the F4 channel, showing detected sleep spindles (in pink) and slow waves (in light blue).

**Table S1 | Number of detected slow waves**

**Table S1 |** NaN indicates removed channels

**Table S2 | Number of detected sleep spindles**

**Table S2 |** NaN indicates removed channels

**Table S3 | Number of detected fast sleep spindles**

**

**Table S3 |** NaN indicates removed channels

**Table S4 | Number of detected slow sleep spindles**

**Table S4 |** NaN indicates removed channels

**Figure S2 | NREM2 sleep EEG power spectra by participant and session**


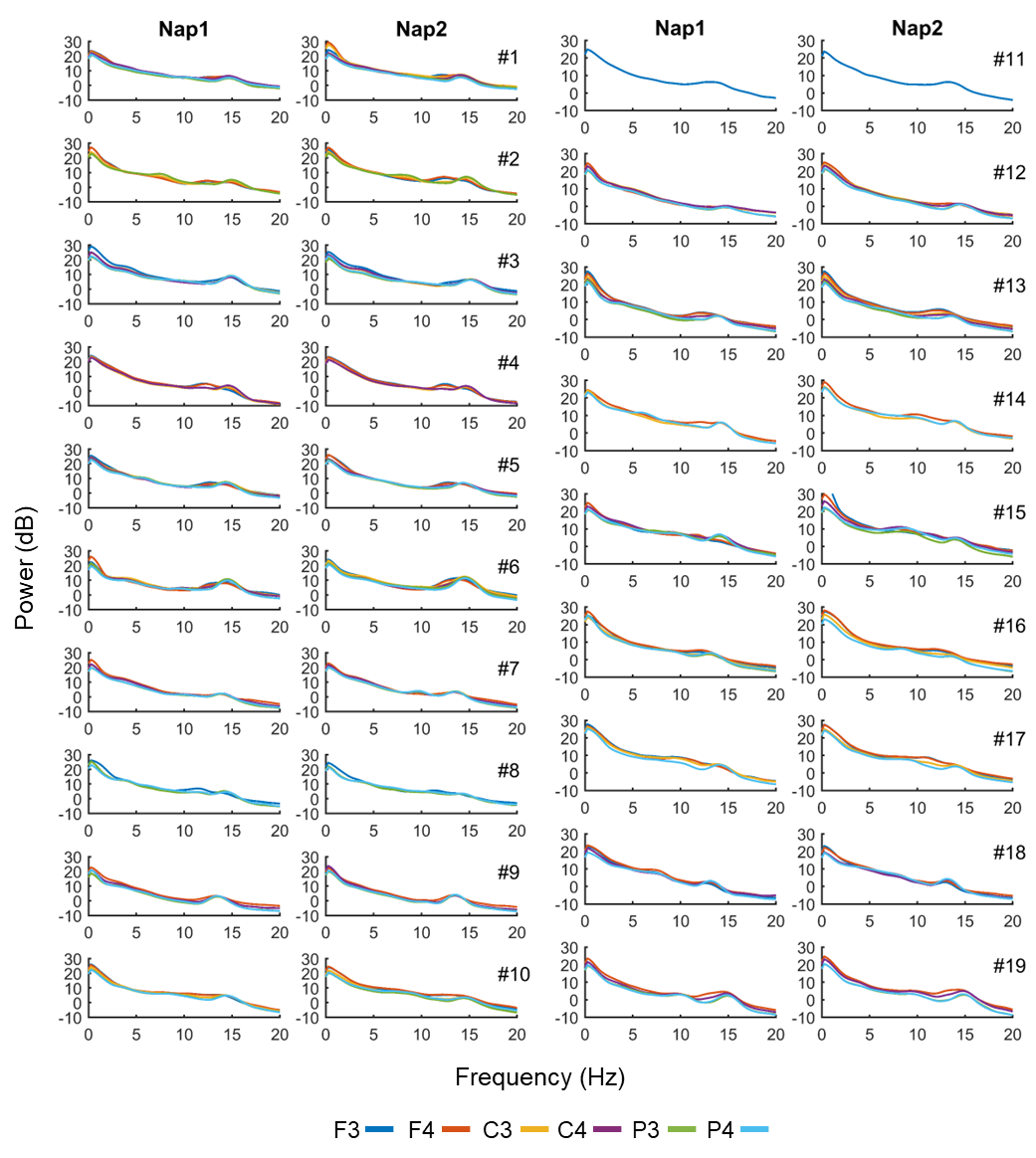


**Figure S2 |** Power spectra are displayed for frontal (F3: dark blue, F4: orange), central (C3: yellow, C4: purple), and parietal (P3: green, P4: light blue) electrodes across the 0-20 Hz frequency range. For each participant, only electrodes retained for analysis are displayed.

**Figure S3 | Spindle-slow wave coupling mean phase differences (Nap1 - Nap2)**

**
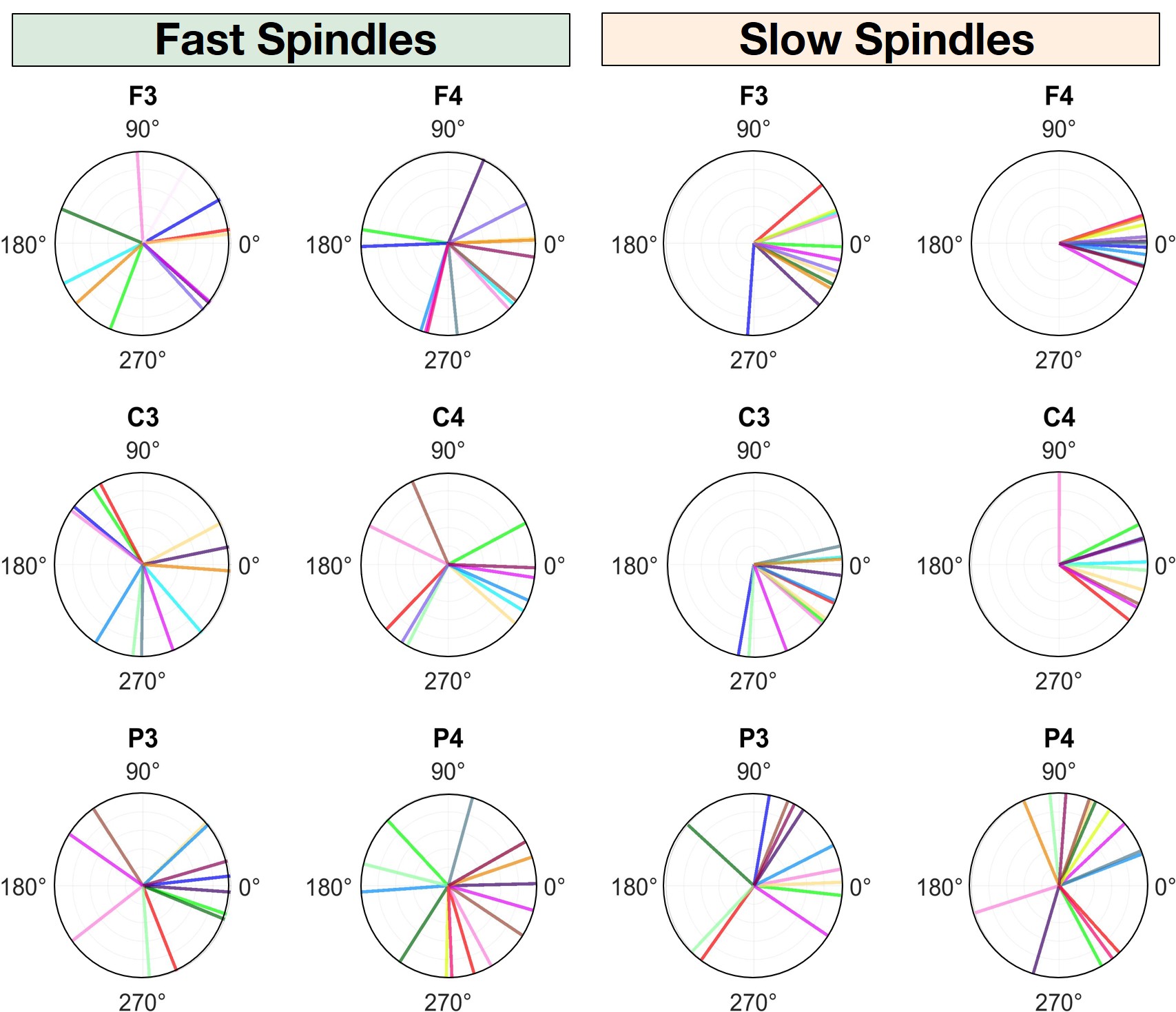
**

**Figure S3 |** Circular plots showing the mean difference in phase coupling of spindles to slow waves between two nap sessions (Nap 1 - Nap 2) during N2 sleep, displayed separately for fast spindles (left panel) and slow spindles (right panel). Each plot corresponds to a specific EEG electrode, and each line represents an individual participant, color-coded consistently across plots. A direction of 0° indicates no change in preferred coupling phase between Nap 1 and Nap 2, whereas angles distant from 0° reflect a shift in coupling phase across sessions.

**Figure S4 | Preferred delta phase (0.5–2 Hz) modulating sigma amplitude (8–13 Hz and 13–18 Hz) within slow wave events across two nap sessions**

*
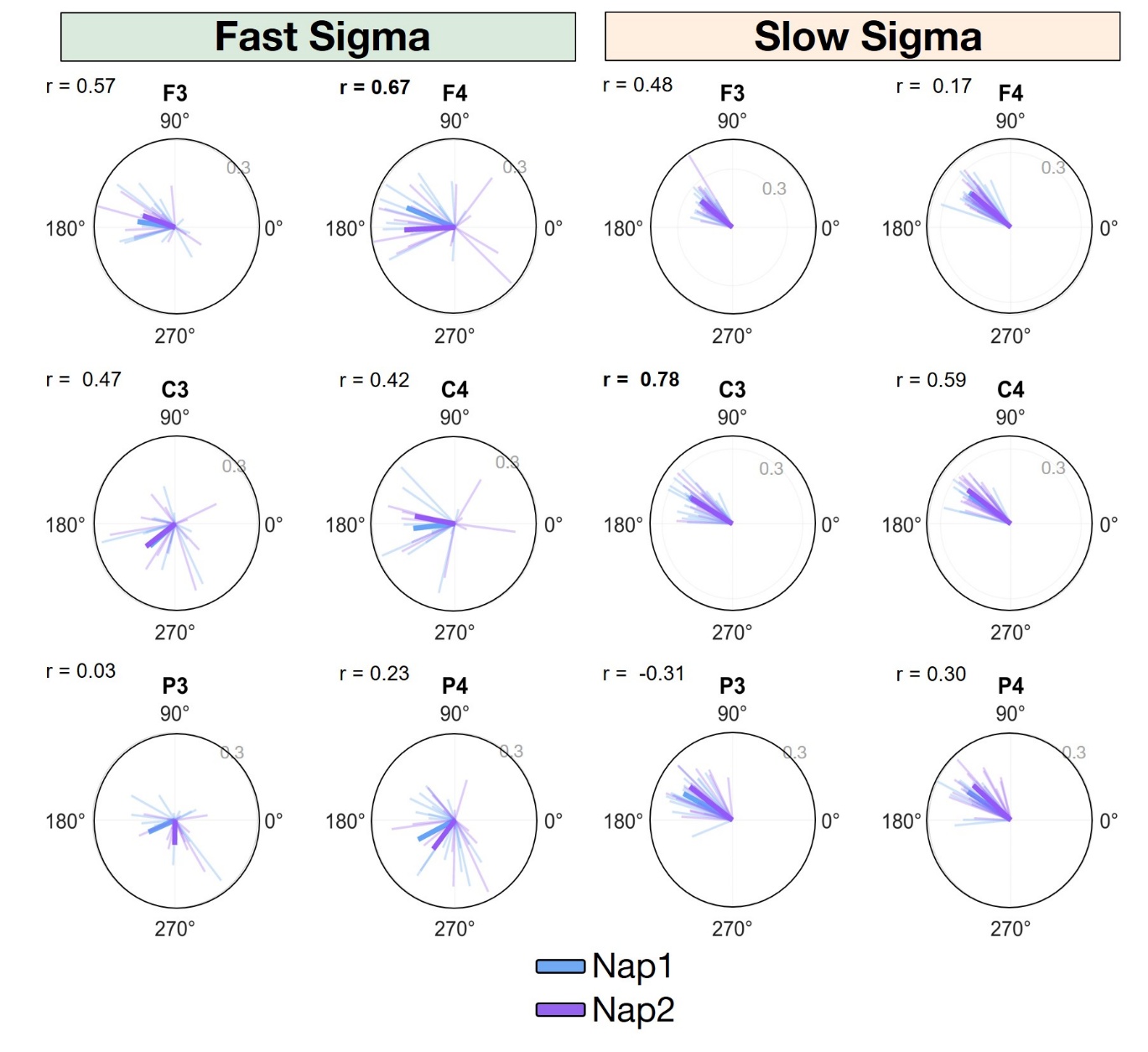
*

**Figure S4 |** Circular plots showing the preferred delta phase (0.5-2 Hz) associated with maximal sigma amplitude (fast: 13-18 Hz and slow: 8-13 Hz) within the detected slow wave events across two nap sessions (Nap 1 in blue, Nap 2 in purple). For each EEG channel, the coupling between delta phase and sigma amplitude was computed separately for fast and slow sigma, considering only the signal between the negative zero-crossing and the endpoint of each slow wave. Vectors represent the circular mean direction and the strength of phase locking (resultant vector length, r) for each participant (shaded colors) and for participants’ mean (dark colors). r: circular correlation coefficient.

**Figure S5 | Temporal dynamics of spindle-slow wave co-occurrence in naps**


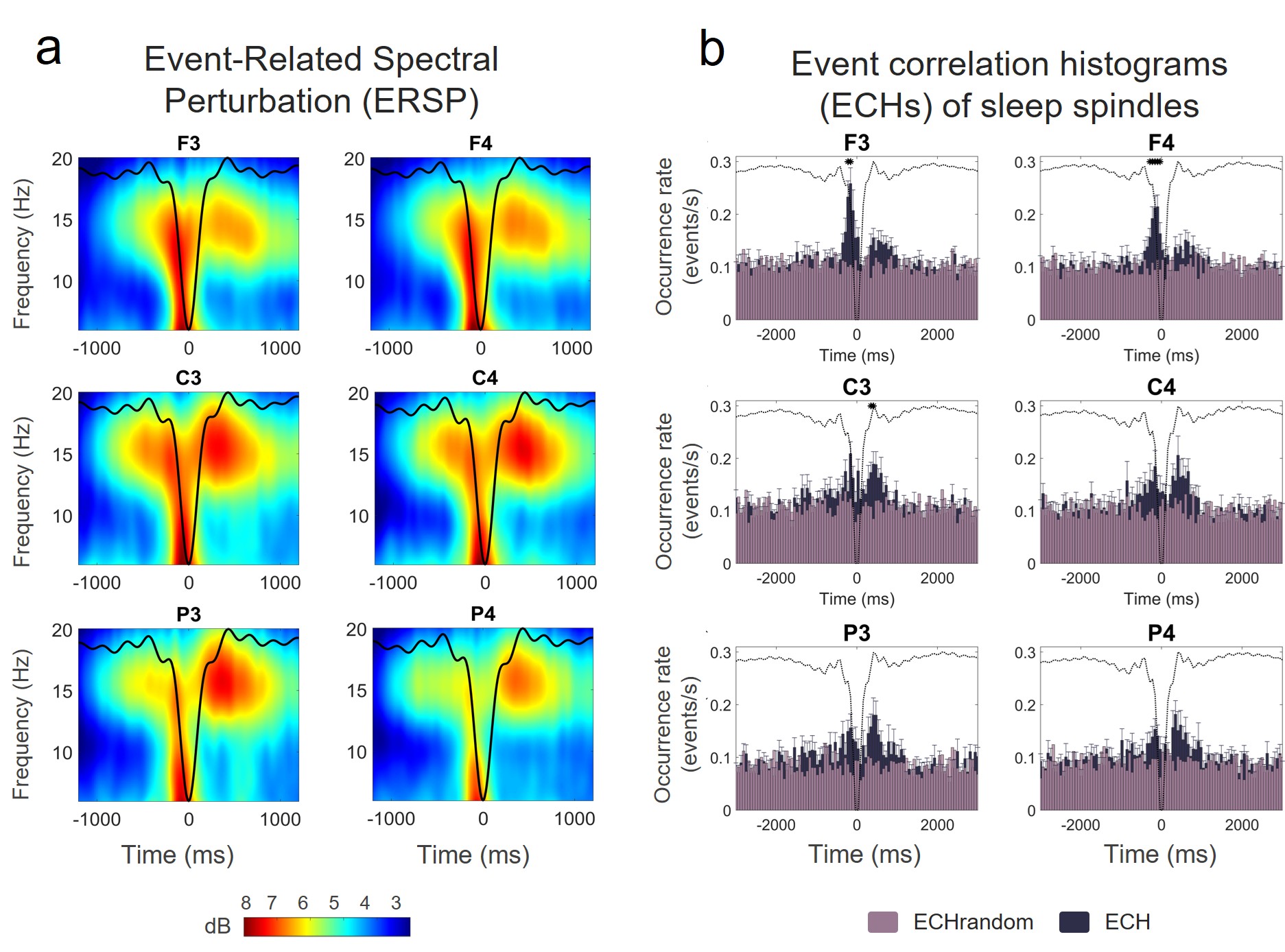


**Figure S5 |** a) Time-frequency plots of event-related spectral perturbation (ERSP) for frontal (F3, F4), central (C3, C4), and parietal (P3, P4) electrodes, combining data from Nap 1 and Nap 2. Time 0 represents the event onset - slow wave negative peak. The black waveform superimposed on each plot represents the average slow wave. ERSP plots show power modulations in decibels (dB). b) Event correlation histograms (ECHs) of sleep spindles around slow wave negative peaks. The histograms illustrate the rate of detected spindle occurrence (events/s) within a ±3-second window centered on the negative peak of the slow wave, with bins of approximately 63 ms. Dark violet bars indicate the mean ECH values with their standard error, while pink bars represent the mean of randomized ECHs (rECHs) obtained by jittering the timing of slow wave peaks within ±3 s, each with corresponding standard error bars. Asterisks above the bars indicate bins with significant differences between observed and randomized data (p < .05), determined through paired-sample t-tests (5000 permutations) and corrected for multiple comparisons using Threshold-Free Cluster Enhancement (TFCE). A black dotted line shows the normalized mean slow wave amplitude for each channel, serving as a temporal reference for spindle occurrences. Data from only six representative electrodes (F3, F4, C3, C4, P3, P4) are displayed.
